# Gorlin-Goltz syndrome: incidental finding on routine ct scan following car accident

**DOI:** 10.1186/1757-1626-2-9087

**Published:** 2009-11-25

**Authors:** Christina Kalogeropoulou, Petros Zampakis, Santra Kazantzi, Pantelis Kraniotis, Nicholas S Mastronikolis

**Affiliations:** 1 Department of Radiology, University Hospital of Patras, Patras, 26500, Greece; 2 Department of Otorhinolaryngology, University Hospital of Patras, Patras, 26500, Greece

## Abstract

**Introduction:**

Gorlin-Goltz syndrome is a rare hereditary disease. Pathogenesis of the syndrome is attributed to abnormalities in the long arm of chromosome 9 (q22.3-q31) and loss or mutations of human patched gene (PTCH1 gene). Multiple basal cell carcinomas (BCCs), odontogenic keratocysts, skeletal abnormalities, hyperkeratosis of palms and soles, intracranial ectopic calcifications of the falx cerebri and facial dysmorphism are considered the main clinical features. Diagnosis is based upon established major and minor clinical and radiological criteria and ideally confirmed by DNA analysis. Because of the different systems affected, a multidisciplinary approach team of various experts is required for a successful management.

**Case presentation:**

We report the case of a 19 year-old female who was involved in a car accident and found to present imaging findings of Gorlin-Goltz syndrome during a routine whole body computed tomography (CT) scan in order to exclude traumatic injuries.

**Conclusion:**

Radiologic findings of the syndrome are easily identifiable on CT scans and may prompt to early verification of the disease, which is very important for regular follow-up and better survival rates from the co-existent diseases.

## Introduction

Gorlin-Goltz syndrome also known as Nevoid Basal Cell Carcinoma Syndrome (NBCCS), was first reported in 1894 [1], but delineated by Gorlin and Goltz [2] in 1960, as a distinct entity consisting of ectodermal and mesodermal abnormalities. This is a hereditary disease with autosomal dominant trait, characterised by high penetration and variable expressiveness, even if sporadic cases have been described. The estimated prevalence varies from 1/57,000 to 1/256,000 among various studies, with a male-to-female ratio of 1:1 [3].

The pathogenesis of Gorlin-Goltz syndrome is attributed to abnormalities linked to the long arm of chromosome 9 (q22.3-q31). It has been reported that loss of human patched gene (PTCH1 gene), which is a tumor suppressor gene, could be the molecular origin of the syndrome [4]. This gene is significant for embryonic structuring and cellular cycle and thus its mutation comprises a key event for the development of the disease. Several different mutations of the *PTCH1 *gene have been identified in patients with NBCCS [5].

Many different signs and symptoms have been described for this syndrome. Main clinical manifestations include multiple basal cell carcinomas (BCCs), odontogenic keratocysts, skeletal abnormalities, hyperkeratosis of palms and soles, intracranial ectopic calcifications of the falx cerebri, and facial dysmorphism such as macrocephaly with frontal bossing, cleft lip/palate and severe eye anomalies. Intellectual deficit is present in up to 5% of cases. Various neoplasms like medulloblastomas, meningiomas, ovarian and cardiac fibromas have been also reported [3,6,7]

Differential diagnosis should be done mainly from a few rare dermatological disorders, like Bazex syndrome, trichoepithelioma papulosum multiplex and Torre's syndrome (Muir-Torre's syndrome) [3].

It is very important to establish diagnosis as soon as possible to prevent fatal consequences, mainly from multiple skin cancers and other tumors associated with the syndrome.

## Case presentation

We report the case of a 19 year-old female who was found to present imaging findings of Gorlin-Goltz syndrome. She was incidentally involved in a car accident and a routine whole body multidetector computed tomography (MDCT) scan was performed in order to exclude traumatic injuries.

**Figure 1:**
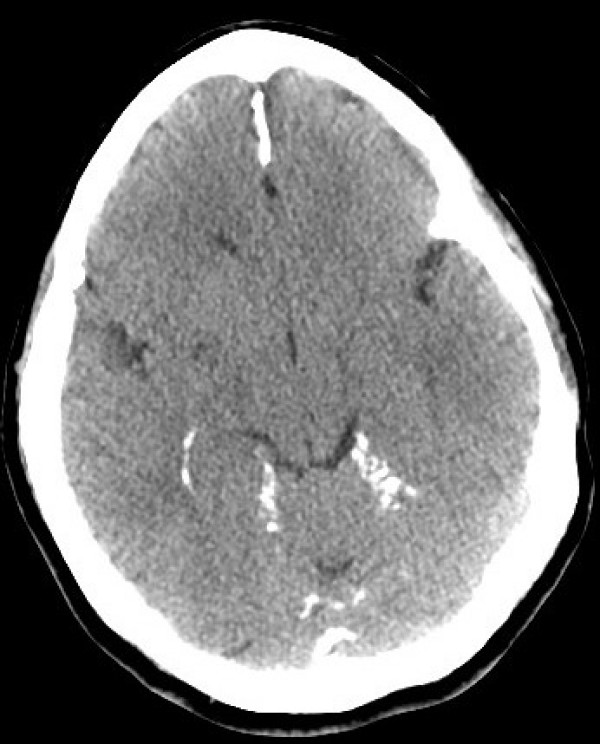
**Unenhanced brain computed tomography scan (Axial view):  Ectopic multiple gross calcifications of falx cerebri are visible**.

**Figure 2:**
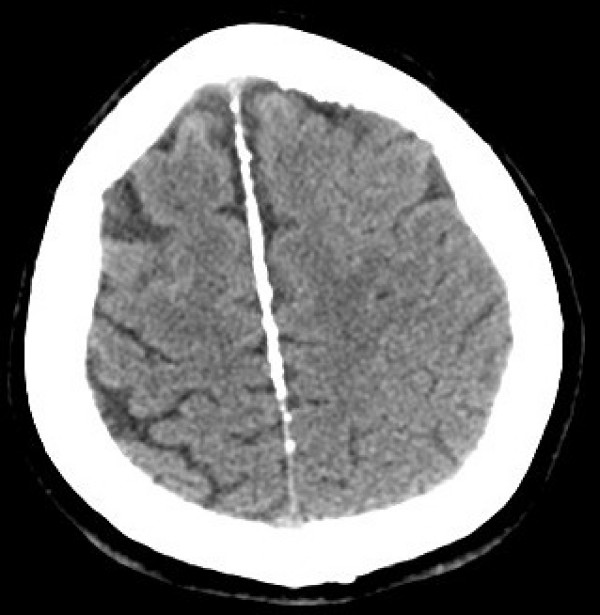
Unenhanced brain computed tomography scan (Axial view): Ectopic multiple gross calcifications of the tentorium are visible.

**Figure 3:**
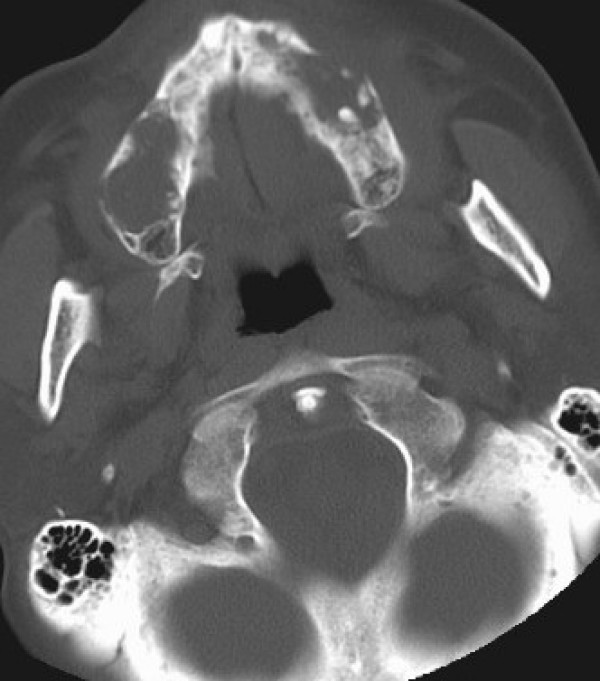
**Facial computed tomography scan (Axial view, bone windows): Multiple odontogenic keratocysts are present in the upper mandible**.

**Figure 4:**
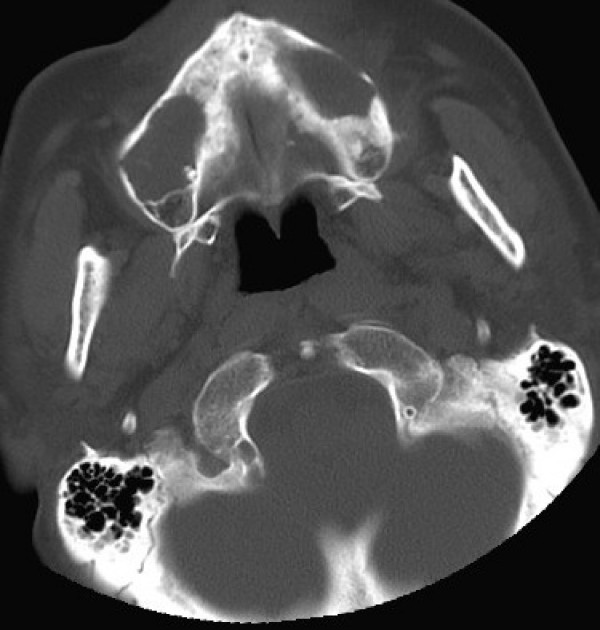
**Facial computed tomography scan (Axial view, bone windows): Multiple odontogenic keratocysts are present in the upper mandible**.

**Figure 5:**
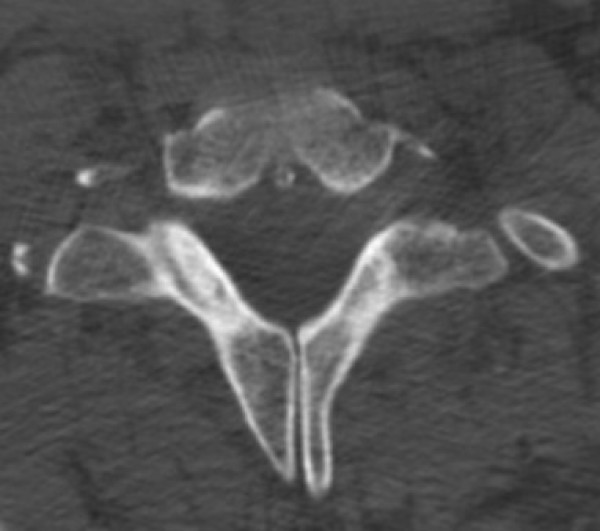
**Axial computed tomography scan at the level of T1 vertebra (bone windows)**. Bifid spinous process of T1 vertebral body is present.

CT scan showed bilamellar calcifications of falx cerebri and tentorium (Figs 1, 2), multiple odontogenic cysts of upper mandible (Figs 3, 4) and vertebral anomalies [bifid vertebrae at T1 level (Fig 5) and at sacral bone].

No skin lesions were detected in the patient. She was discharged since no traumatic lesions were detected but a clinical examination was scheduled for the syndrome follow up evaluation, since the establishment of the diagnosis was made from the radiological investigation. Furthermore, a DNA analysis was advised, in order to confirm the diagnosis in the gene level.

## Discussion

Early diagnosis of Gorlin-Goltz syndrome is important due to susceptibility of affected people to multiple neoplasms in early age. UV exposure should be avoided because of increased susceptibility to basal cell carcinomas. As a consequence, diagnosis of Gorlin Goltz syndrome at an early age could alert affected people to more often and detailed routine check-up in order to prevent cancer occurrence or to treat variable abnormalities [8].

Evans et al [9] first established major and minor criteria for diagnosis of this rare entity, later modified by Kimonis et al [10]. Although these criteria are not absolute, they can help guiding the clinician's laboratory evaluation for both diagnostic purposes and routine follow up.

Major criteria consist of:

I. More than two basal cell carcinomas or one in patient <20 years old

II. Odontogenic keratocysts of the jaw

III. Three or more palmar or plantar pits

IV. Bilamellar calcifications of falx cerebri and tentorium

V. Bifid or fused, or markedly splayed ribs

VI. First-degree relative with Gorlin-Goltz syndrome.

Minor criteria consist of:

I. Macrocephaly,

II. Congenital anomalies (cleft lip or palate, frontal bossing, coarse facies, and moderate or severe hypertelorism)

III. Other skeletal anomalies (Sprengel deformity, marked pectus deformity, and marked syndactyly of the digits)

IV. Radiologic anomalies (such as bridging of the sella turcica, vertebral anomalies, modelling defects of the hands and feet, or flame-shaped lucencies of the hands and the feet

V. Ovarian fibroma or myeloblastoma.

Two (2) major criteria or 1 major and 2 minor criteria are obligatory in order to diagnose Gorlin-Goltz syndrome [10].

In our case, two major criteria (odontogenic keratocysts of the jaw and calcification of falx-cerebri and tentorium) and one minor [skeletal anomalies (bifid vertebraes)] were detected.

By definition the radiologists could play an important role in the diagnosis of this rare entity, because several major and minor criteria are detected only after a radiological exam, however the syndrome is not well known among them.

Therefore high suspicion is mandatory concerning detection of odontogenic keratocysts or calcifications of falx cerebri and tentorium, especially at young patients since the syndrome is mostly known among dentists. These easily detectable abnormalities in CT scans, can prompt further chromosomal diagnostic work-up to confirm the diagnosis.

Several imaging modalities can be used for the detection and investigation of Gorlin-Goltz syndrome. Skull radiography shows calcifications of the falx cerebri, tentorium cerebelli and diaphragma sellae, while skeletal X-ray reveals bifid, hypoplastic, fused, partially missing, or splayed ribs in 38-60% of patients with the 3rd, 4th and 5th ribs most commonly affected. Other imaging findings of the syndrome are malformations at the occipitovertebral junction, cervical or upper thoracic vertebral fusion and small, pseudocystic, lytic bone lesions known as "flame-shaped lucencies" on radiographs of the hands (30%) and feet (17%) of affected individuals.

Odontogenic keratocysts, which are relatively common in Gorlin-Goltz syndrome, are diagnosed with dental panoramic radiography. Keratocysts may show a uni- or multilocular pattern and the cystic spaces may have a smooth or scalloped border [3]. Cardiac echocardiography may be used to look for cardiac fibroma (relatively rare). Brain MRI may be less sensitive than plain radiography for calcification, and it may be indicated if medulloblastoma is suspected in a child with the syndrome. Finally, pelvic ultrasound is the modality of choice for ovarian fibromas.

Early recognition of the disease, a detailed family history and a thorough evaluation of signs and symptoms are the cornerstones for an appropriate management. Because of the different systems affected and the diversity in clinical picture, once diagnosis is established, a multidisciplinary approach team of various specialists is required for a successful treatment. A dermatologist should maintain ongoing surveillance and treatment of BCCs, thus planned visits are recommended to identify and treat lesions when they are as small as possible [8]. Surgical excision, laser ablation, photodynamic therapy and topical chemotherapy are recommended treatment options, while radiotherapy should be avoided. Vitamin A analogs may play a preventive role against new skin cancers growth [8]. Frequent dental visits are also obligatory. Jaw keratocysts are often recurrent in up to 60% and demand repeated surgical excisions. A neurologist should also play a role, because 5-10% of the patients may develop brain medulloblastoma, which comprises a potential cause of early death. Survival in Gorlin-Goltz patients is not noticeably altered, however morbidity from complications can be considerable [9]. Nowadays gene mutation analysis, if feasible, can confirm diagnosis. Antenatal diagnosis is possible with ultrasound scans and DNA analysis extracted from fetal cells after amniocentesis or chorionic villus sampling. Thus, a genetic counsellor is a critical component of the ongoing care of the patient, especially when fertility issues arise. As new research is performed, the availability, sensitivity, and specificity of molecular testing may further improve [3,8].

## Conclusion

Our case highlights the importance of the awareness of this rare syndrome especially in young people without any skin lesions. We have seen that it is useful to keep in mind the existence of the syndrome and recognize the presence of some major criteria (odontogenic cysts at the jaw or calcifications of falx cerebri and tentorium) as these are easily recognizable in CT scan of the head and neck thus establishing the diagnosis, even as an incidental finding offering the opportunity for frequent follow-ups and therefore increasing the chances for better overall survival rates.

## Consent

Written informed consent was obtained from the patient for publication of this case report and accompanying images. A copy of the written consent is available for review by the Editor-in-Chief of this journal.

## Competing interests

The authors declare that they have no competing interests.

## Authors' contributions

NSM is the referring doctor for patient's follow-up and co-ordinates other sub-specialities for that reason. He also focused on the clinical relevance of this rare entity. CK, PZ, SK and PK analyzed and interpreted the radiologic findings, as well as they found all the relevant information regarding this relatively rare entity. CK, SK, PZ, PK were also major contributors in writing the manuscript, focused on the radiologic findings, while NSM focused on the clinical relevance. All authors read and approved the final manuscript.
